# Recent biotechnological applications of value-added bioactive compounds from microalgae and seaweeds

**DOI:** 10.1186/s40529-024-00434-y

**Published:** 2024-09-23

**Authors:** Salma N. Eladl, Aya M. Elnabawy, Eladl G. Eltanahy

**Affiliations:** Algae Biotechnology and Water Quality Lab, Faculty of Science, Mansoura University, Mansoura, 35516 Egypt

**Keywords:** Seaweeds, Microalgae, Polysaccharides, Biofertilisers, Nanoparticles, Vitamins

## Abstract

Microalgae and seaweed have been consumed as food for several decades to combat starvation and food shortages worldwide. The most famous edible microalgae species are *Nostoc*, *Spirulina*, and *Aphanizomenon*, in addition to seaweeds, which are used in traditional medicine and food, such as Nori, which is one of the most popular foods containing *Pyropia* alga as a major ingredient. Recently, many applications use algae-derived polysaccharides such as agar, alginate, carrageenan, cellulose, fucoidan, mannan, laminarin, ulvan, and xylan as gelling agents in food, pharmaceuticals, and cosmetics industries. Moreover, pigments (carotenoids particularly astaxanthins, chlorophylls, and phycobilins), minerals, vitamins, polyunsaturated fatty acids, peptides, proteins, polyphenols, and diterpenes compounds are accumulated under specific cultivation and stress conditions in the algal cells to be harvested and their biomass used as a feedstock for the relevant industries and applications. No less critical is the use of algae in bioremediation, thus contributing significantly to environmental sustainability.

This review will explore and discuss the various applications of microalgae and seaweeds, emphasising their role in bioremediation, recent products with algal added-value compounds that are now on the market, and novel under-developing applications such as bioplastics and nanoparticle production. Nonetheless, special attention is also drawn towards the limitations of these applications and the technologies applied, and how they may be overcome.

## Background

Algae are macroscopic and microscopic photosynthetic organisms that have grown in prominence recently due to their potential as a renewable and sustainable source for various products. They can grow either autotrophically, heterotrophically, or mixotrophically. However, the autotrophic growth mode is predominant among algae (Patel et al. 2019). Their ability to be cultured on water or waste and their rapid growth make algae a promising alternative for various products, saving time, land, and costs (Cheah et al. 2016). During the metabolic process of algal development, a diverse range of primary and secondary metabolites are produced from macro and microalgae (Chan et al. 2022). Among the most promising uses of algae is creating products with added value. Although the biochemical composition of algal cells may be similar to some higher plants and fungi, many microalgae, in addition to seaweeds, can grow in saline aquatic environments and can be cultured in arid areas or areas containing well water with a high salinity that makes it unsuitable for traditional agriculture in addition to its high growth rates compared to higher plants, which is of great importance in using these algae to fill the strategic gap in food and energy alike.

Different algae components, such as pigments, lipids, proteins, and carbohydrates, are extracted, purified, and processed to produce these goods. Yet, microalgae are more explicitly studied than macroalgae, given their high profile of amino acids, lipids, and proteins (Koyande et al. 2019; Kumar et al. 2020). Anyhow, mainly marine macroalgae make an immense source of polysaccharides and polysaccharides-based industries (Rajendran 2020). Algae-derived products with added value can be used in various industries, including food, cosmetics, pharmaceuticals, and biofuels. Due to their high nutritional value and bioactive properties, algae-derived ingredients are utilised to produce nutraceuticals, supplements, and functional foods. For instance, algae’s carotenoids, phycobilins, and polyunsaturated acids show neuroprotective, anti-ageing, antibacterial, and anticancer properties (Galasso et al. 2019). Moreover, algal lipids and carbohydrates can be used in biofuel industries (Goswami et al. 2020). Biofertilisers based on algae proved efficient in improving soil fertility, crop yield, and bioavailability due to the presence of vitamins, minerals, as well as considerable amounts of proteins and carbohydrates in their biomass (Alvarez et al. 2021; Sarma et al. 2021). Also, recent studies have shown the potential of algal biomass in the production of nanoparticles and bioplastics.

To elaborate, many known common algae, such as *Spirulina* alga, are used in different applications. The name *Spirulina* is derived from the spiral, filamentous, and microscopic nature of the spiral cyanobacteria. It is used as human food and considered one of the most important sources of vitamins, especially provitamin A (beta-carotene) and B12, proteins up to 70%, tocopherols, phenolic and linolenic acids, and contains a percentage of minerals, especially iron and both intracellular and extracellular polysaccharides (Liu et al. 2023). Digestion of *spirulina* is effortless due to the lack of cellulose cell walls. This alga belongs to substances that have been declared safe by the US Food and Drug Administration, given that numerous scientific studies have demonstrated the its safety and only spreads in lakes with a high degree of alkalinity under controlled conditions, making it easy to grow without contaminants. It is usually offered at the healthy food providers and is consumed through drinks and tablets as supplements without side effects (Torky et al. 2023).

Another example is *Aphanizomenon flos-aquae* (AFA), blue-green unicellular prokaryotic microalgae belonging to the Cyanophyta phylum. It works as a significant oxygen source at the planet Earth’s beginning (Hamilton et al. 2016). AFA is found in many places of leafy green algae and cyanobacteria and is known as Klamath algae because it is common in Klamath Lake, North America as well as in the surrounding areas. The blue-green colour of AFA gives phycocyanin pigment up to 15% of the organism’s dry weight. This pigment contains many anti-inflammatory effects, as well as anti-oxidants. Through previous studies, it has been shown that a natural extract of AFA enriched with phycocyanin protects blood plasma samples and also works to protect normal human erythrocytes from the process of oxidative damage in-vitro (Risuleo and La Mesa 2021).

*Nostoc* is also a genus of microscopic algae (Cyanobacteria) found as clusters of moss floating in water and soil habitats containing chlorophyll, phycoerythrin, and phycocyanin, and can tolerate harsh conditions like drought. Heterocysts in *Nostoc* spp. are responsible for nitrogen fixation (Gademann and Portmann 2008). Many species, *N. flagelliform*, *N. sphaeroides*, and *N. muscorum*, are collected from natural habitats and consumed by the Chinese as food for several decades (Lopes et al. 2022). Moreover, *Nostoc* is used as a health food worldwide due to its therapeutic and physiological activities, besides its immuno-regulating, anticancer, anti-inflammatory and antioxidant properties. Furthermore, compounds like cytotoxic cryptophytes, nostocyclopeptides, antiviral cyanovirin-N, and hepatotoxic microcystins (Fidor et al. 2019) were also detected in *Nostoc*. Also, microginins, cyanopeptolins, and anabaenoptins are biologically active *Nostoc* peptides that inhibit potent proteases. Unfortunately, due to the increased demand and the subsequent use of pesticides and fertilisers, the natural population for these species has decreased (Lopes et al. 2022).

Macroalgae are nonetheless important than their micro counterparts. One of the most significant seaweed species used primarily for food and functional food worldwide is *Pyropia* spp., a red edible macroalga (Kim et al. 2017; Park et al. 2023). The two Pyropia species that are most frequently used are *Pyropia yezoensis* and *Pyropia haitanensis*(Xie et al. 2013). Thus, many value-added bioactive substances, including proteins, fatty acids, minerals, vitamins, polysaccharides, and pigments, are found in *Pyropia* species (Bito et al. 2017). These bioactive substances have anti-inflammatory, antiviral, immunomodulating, anticancer, anticoagulant, antioxidant, hypotensive, antihypertensive, antihypertensive, and anti-ageing properties (Bhatia et al. 2008). For instance, the polysaccharides from *Pyropia*, and especially porphyran, have immunoregulatory, antioxidant, and anticancer effects (Isaka et al. 2015; Cao et al. 2016; Venkatraman and Mehta 2019). To provide more specifics, porphyran from *Pyropia* has an antioxidant action by suppressing nitric oxide and tumour necrosis factor. It also has a lower molecular size than typical porphyran (Yanagido et al. 2018). *Pyropia* can produce agar for various food and research purposes in addition to health-based bioactivities (Yanagido et al. 2018). Additionally, *Pyropia* can create a violet natural food colour (Kohata et al. 2010).

## Algal bioactive products

Microalgal cells and seaweeds do not precisely have the same contents for the same species. However, it may change dramatically due to climatic changes in open environments and laboratory scales. Therefore, scientists found that using different food media and lighting intensity, exposing the organism to varying proportions of light and dark daily, and even starving it of one of the macro elements may significantly affect the natural products produced inside its cells. Therefore, some of these experiments and variables have been recorded in Table 1. The most important of these products and the methods of stimulating their production will also be discussed in detail.

**Table 1:** Algal strains, growth medium, incubation conditions, biomass, and produced products

Algal strain	Medium	Light intensity (Light: Dark)	Duration (days)	Biomass Dry Wt.	Product %	Notes	References
*Nostoc* sp	liquid nutritive medium, BG11 and BG110 (No sodium nitrate) media	40 µmol m^− 2^s^− 1^ (16 h:8 h)		361 mg L^− 1^	bioactive peptides (e.g. Cyanopeptolins inhibit serine proteases; Cyanovirin-N that act as antiviral agent)	Modifications in light uptake and electron transport proteins	El-fayoumy et al. (2021) Fidor et al. (2019) Canonico et al. (2020) (Mazur-Marzec et al. 2018) (Botos and Wlodawer 2003)
*Spirulina* sp	Selective liquid medium viz. Zarrouk medium	60 µmol m^− 2^s^− 1^ (12 h:12 h)		(0.177, 0.170 mg ml^− 1^)	Protein 50–60% (Phycocyanin and Allophycocyanin 15–20% that act as antioxidant, anti-inflammatory, and immune-enhancing agents, and used as probes)	Moisture had a significant effect on the efficacy of the process. Optimal conditions: 98.8% relative humidity	Madkour et al. (2012) Michael et al. (2019) Devanathan et al. (2016) Pelizer et al. (2015) (Rito-Palomares et al. 2001)
*Porphyra umbilicalis*	Filtered Sea Water	140 µmol m^− 2^ s^− 1^ (12 h:12 h)			Carrageenan (19.9%) Pectin (19.2%) Proteins (15%)	Multicomponent fractionation to recover proteins and polysaccharides more efficiently	Wahlström et al. (2018)
*Dunaliella salina*	Johnson mixotrophic media	60 µmol m^-2^s^-1^ (24 h: 0 h)	21	1.015 g L^-1^	Carotenoids (0.8%)	Optimisation of glucose, nitrate and phosphate levels induced biomass and β-Carotene production	Morowvat and Ghasemi (2016)
*Nannochloropsis salina*	Guillard’s medium in artificial seawater	150 µmol m^-2^^-1^	14	0.18 g L^-1^	Fatty acids (70%)	Higher levels of Total Fatty Acids at 17 °C and under nitrate starvation	Hoffmann et al. (2010)
*Phormidium* sp.	BG-11 + 25 g L^− 1^ sea salt	44 µmol m^− 2^s^− 1^ (24 h: 0 h)	12	1.22 g L^− 1^	Protein (38.3%) Phycobiliproteins (19.38%)	NH_4_Cl as a nitrate source is more effective than NaNO_3_	Khazi et al. (2018)
*Pseudoscillatoria* sp.	BG-11 + 25 g L^-1^ sea salt	44 µmol m^− 2^^− 1^	12	1.12 g L^-1^	Protein (43.2%) Phycobiliproteins (19.99%)	NH_4_Cl as a nitrate source is more effective than NaNO_3_	Khazi et al. (2018)
*Anabaena cylindrica*	Modified ASM-1 medium (MLA)	320 µmol m^-2^s^-1^ (12 h:12 h)	14	0.65 g L^-1^	Protein (e.g. phycoerythrins, phycocyanins, and allophycocyanins) (68.6%) Vitamin K_1_ (0.02%)	Higher light intensity led to a rise in growth rate while Vitamin K_1_ increased with increasing nitrates	Tarento et al. (2018)
*Haematococcus pluvialis*	Bold’s Basal medium (BBM)	150 µmol m^− 2^s^− 1^	4–6		Astaxanthin (2–3%)	The addition of trace elements and B vitamins to MM1, MM2, and KM2 media resulted in 1.5, 1.35, and 2.0-fold increases in astaxanthin content, respectively	Tran et al. (2019)
*Gelidium pulchellum*	Modified PES medium	10–430 µmol photons m^− 2^ s^− 1^	3–4		Agar (31–38.6%)	After filtration, agar was obtained via the freeze-thaw method and dehydrated with 96% ethanol before desiccating at 60^o^ C	Sousa-Pinto et al. (1999)

Advances in cultivation methods include customised media composition for the respective algal species documented in the above table. Examples may include the optimization of carbon sources for lipid accumulation in *Nannochloropsis salina* or increasing nitrogen concentrations, which enhance protein production in *Spirulina* (Borowitzka 2013). Moreover, shifting towards mixotrophic conditions results in increased metabolite production including bioactive compound yields (Mata et al. 2010). Environmental factors such as light intensity, wavelength, temperature, and pH are also important factors, for example, exposing *Porphyra umbilicalis and Dunaliella salina* to certain light wavelengths induces the production of phycobiliproteins and carotenoids respectively. Additionally, improvements in cell disruption methods have facilitated extraction. For example, in ultrasonication, the application of high-frequency sound waves has been applied in extracting pigments and lipids (Lee et al. 2010). Also, enzyme-assisted methods are applied in extracting sensitive compounds, for example, in the degradation of the cell walls with protease and cellulase (Postma et al. 2015). Similarly, modern extraction techniques using solvents like microwave-assisted extraction and supercritical fluid extraction are involved in the processes of extracting fatty acids and carotenoids at very high yields and purity (Plaza et al. 2008). These technological developments enhance production efficiency and make cost-effective, sustainable, high-value bioactive compound extraction from the diversity of algal species.

### Polysaccharides

#### Agar

**Fig. 1:**
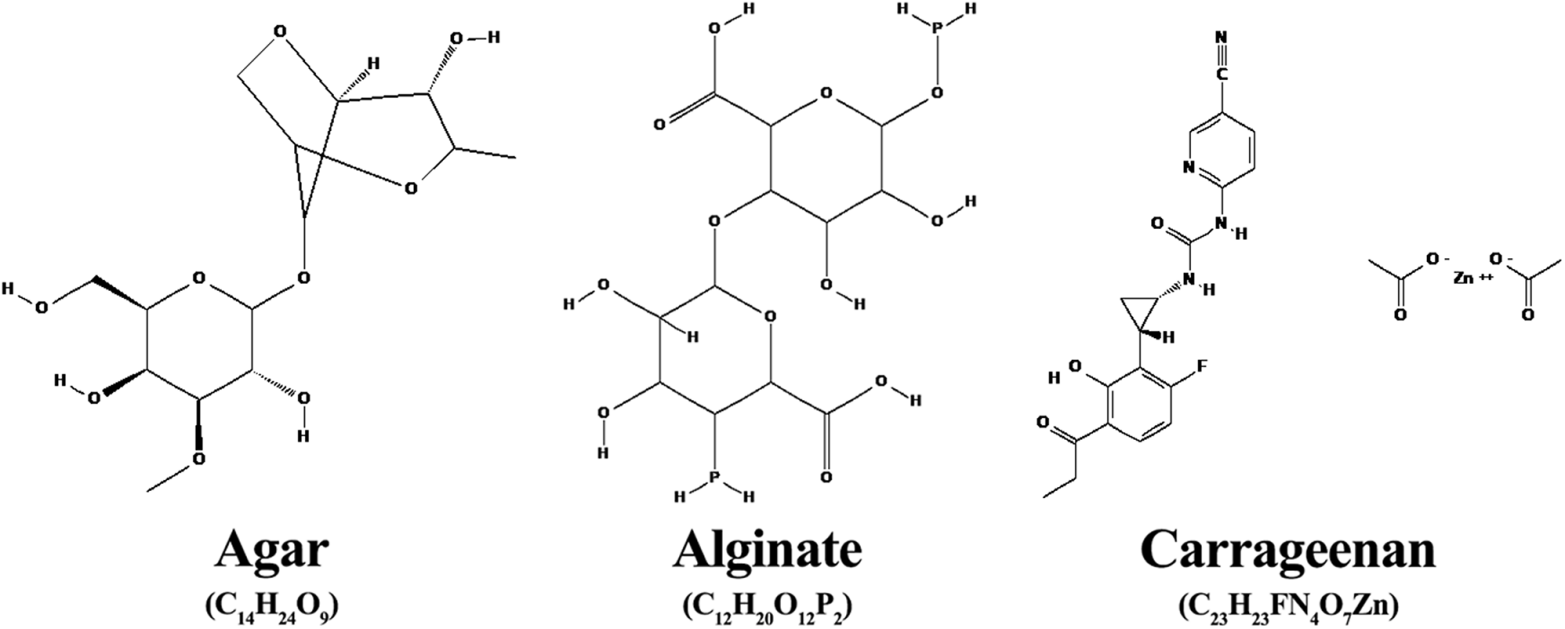
Chemical structures of agar, alginate, and carrageenan

Agar originates back to 1658 in Japan. It is mainly made up of sulfated galactan (Fig. 1), which is easily soluble in water. Agar can be found in many red algae as well as vegetable cells and characterised by its flexibility and mobility, it can adapt to diverse marine environments (Cebrián-Lloret et al. 2024). Agar is utilised in many applications, including cultivation media for microorganisms, plant tissue transplantation, and insect food preparation (Sousa et al. 2021). Also, agar-based drug delivery systems are developed to treat respiratory disease (Gupta et al. 2023). Additionally, it is used in dental gels to treat teeth, in the cloning of fingerprints for police investigations, and in the reproduction of archaeological remains. Scientists reported that agar-treated mice’s antioxidant enzymes and ageing proteins were regularly controlled compared to control and D-gal-induced groups (Reshma et al. 2022). Also, the agar extracted from *Laminaria digitata* showed protective effects in D-gal-induced mice, including antioxidant and antiaging properties compared to untreated groups.

#### Alginate

Alginates are polysaccharides that contain alternating β-D-mannuronic (M) and α-L-guluronic (G) acid units (Fig. 1) (Galus and Lenart 2013). They can be synthesised from either bacterial or algal sources (Gheorghita Puscaselu et al. 2020). Algal alginates, however, are more frequently used for commercial purposes. The most typical sources of alginate are the algae *Macrocystis pyrifera*,* Ascophyllum nodosum*,* Laminaria* sp., *Sargassum* sp., and *Eclonia maxima*. Alginates are currently used in various industries, including the culinary, pharmaceutical, and medical fields. For instance, sodium alginates are utilised as edible coatings and films in the food sector to protect products against microbiological and physical damage (Bourtoom 2008). Moreover, with the possibility of getting integrated with preservatives, antioxidants, dyes, and additives, alginates can improve the product’s quality and appearance and extend its shelf life (Hammam 2019). Recent research focuses on the applications of alginates in the medical and pharmaceutical fields. Alginate was used as a scaffold for wound healing by mixing at different ratios with gelatin by a method known as freeze-gelation (Afjoul et al. 2020). The scaffold aided in healing wounds by regenerating skin appendages and hair follicles and synthesising collagens. Also, alginate hydrogel used as a carrier for cianoside facilitated the reduction of inflammation and allergic reactions in the skin of mice (Szekalska et al. 2020). Alginates extracted from *Sargassum polycystum* are utilised as gelling agents in the formulation of vitamin C serum gel (Purwanto 2023). Many other trials of using alginates as scaffolds and drug delivery systems have taken place through the formulation of alginate bio-aerogels, wound, foam, and hydrogel dressings, as well as alginate microbeads (Gheorghita Puscaselu et al. 2020). Furthermore, alginate showed protective effects on probiotics by acting as encapsulation to those cells. What is more, they can also be used to manage and control diabetes and obesity (Murakami et al. 2023).

#### Carrageenan

Rhodophyta, particularly Florideophyceae, comprise linear sulfated polysaccharides known as carrageenans (Fig. 1) (Campo et al. 2009). Carrageenans are used as a thickening and gelling agent in the food industry, mainly in producing dairy, jellies and desserts, and processed meat, which gives them an improved texture, proper appearance, and aids in processing (Hotchkiss et al. 2016). A system in which carrageenan microgels were mixed with alginate microgels in a ratio of 1:1 was developed and proved effective in controlling the levels of lipids in the blood and the hormone-releasing process (Chen et al. 2018a). Carrageenan has also been used over the years to clean industrial effluents by co-immobilising microbial cells with Kappa-carrageenan (κ-carrageenan) (Necas and Bartosikova 2013). However, recent work is more focused on the role of carrageenan in the medical and pharmaceutical fields due to its high viscosity, biocompatibility, antimicrobial properties, and high molecular weight (Pacheco-Quito et al. 2020). Iota-carrageenan from *Agardhiella ramosissima* moderated the inflammation in arthritis conditions (Rodrigues et al. 2023). In an in-vitro research study on bone tissue engineering, incorporating whitlockite, carrageenan nanocomposite hydrogel, and a proangiogenic agent accelerated bone repair by enhancing angiogenesis and osteogenesis (Yegappan et al. 2019). In the pharmaceutical industry, carrageenans are utilised as thickeners, binders, coatings, and controlled drug release systems, which is the most significant application (Pacheco-Quito et al. 2020). Additionally, intratumoral injection of λ-carrageenan stopped the growth of tumours in mice with mammary and melanoma tumours, and it improved the immune response to tumours by increasing the release of dendritic cells, activated CD4 + CD8 + T lymphocytes, and tumour-infiltrating M1 macrophages (Luo et al. 2015).

#### Cellulose

**Fig. 2:**
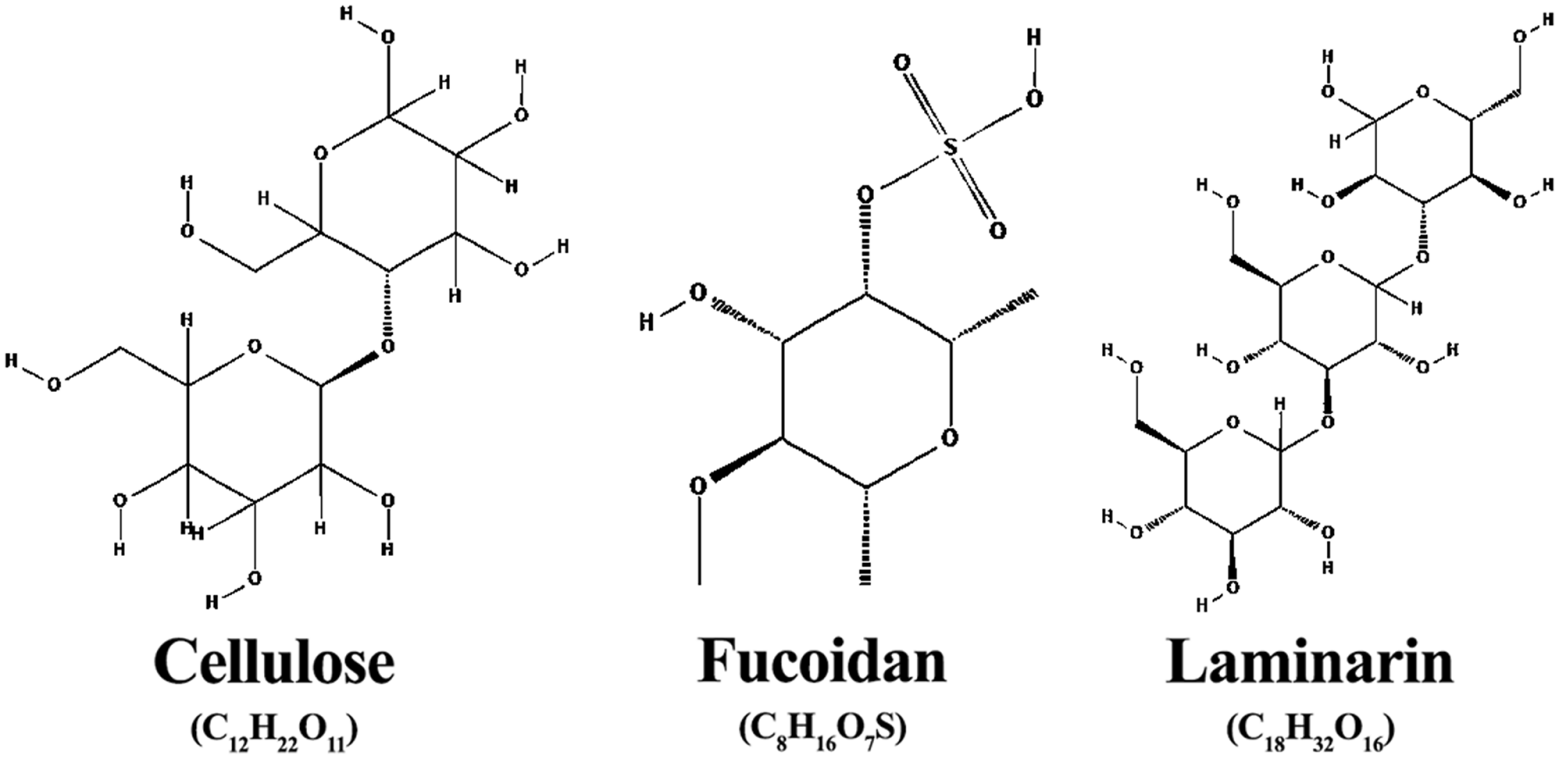
Chemical structures of cellulose, fucoidan, and laminarin

Algal cell walls are composed of a linear chain of many β (1→ 4) linked d-glucose units known as cellulose (Fig. 2) with a dry weight of 9–34% of the cell wall, and other polysaccharides mannan, galactan, carrageenan, agar, and xylan (Baghel et al. 2021). Algal cellulose is similar in many ways to plant cellulose; hence, it can be used as an alternative to plant-origin cellulose, given the more accessible and more efficient extraction process (Hafid et al. 2021). However, algal cellulose has a surface area of about 100 times larger than plant cellulose (Marsin and Tomasz 2005). Cellulose from macroalgae is extensively used in bioethanol production; however, it can also be used as a feedstock to produce many compounds such as bioplastic, paper and pulp, nanocellulose, carboxymethyl cellulose, and microcrystalline cellulose (Baghel et al. 2021). Nanocellulose and carboxymethyl cellulose, alone, have a wide variety of applications in the food, biomedical, and pharmaceutical sectors (Ahmad et al. 2014; Lin and Dufresne 2014). Moreover, nanocellulose extracted from *Ulva lactuca* proved efficient as an antimicrobial polymer (El-Sheekh et al. 2023). Further, it has reportedly been used in developing sensors and electronics (Baghel et al. 2021). Paper produced from *Gelidium corneum* and *Gelidium amansii* cellulose showed superiority over wood-based paper in its opacity and smoothness, which are critical in the quality of paper production (Seo et al. 2010). Bioethanol from enzymatic hydrolysis and fermentation of cellulose from *Ulva* spp. dry biomass was reported (Trivedi et al. 2016). Further, biopolymers from cellulose extracted from freshwater macroalgae like *Cystosphaera jacquinottii* and *Cladophora glomerata* were developed and used in drug delivery, food production and cosmetics applications (Dang et al. 2022). For instance, a biomaterial with crystallinity above 70%, a diameter of 32 nm and high porosity was successfully produced by the alkaline treatment, bleaching and freeze-drying of the cellulose extracted from *Cystosphaera jacquinottii*(Paniz et al. 2019).

#### Fucoidan

Phaeophyceae, or brown seaweeds, are the primary source of fucoidans, a group of sulfated polysaccharides made up of sulfate and L-fucose (Fig. 2) (Etman et al. 2020). Fucoidan, found in brown macroalgae’s cell walls, exhibit antiallergic, antitumour, antioxidant, antimicrobial and anti-inflammatory properties, as well as nano drug delivery systems that make it effective in health, food, and feed applications (Abdel-Latif et al. 2022; George and Shrivastav 2023). Moreover, due to its previously mentioned properties, fucoidan can act as a toxicity modulator, growth promotor, and antimicrobial agent in aquaculture. The antiviral effects of fucoidan were shown when fucoidans from *Laminaria cichorioides* reduced the cells infected with hantavirus from 27.0 to 5.3 (Pavliga et al. 2016). Further, fucoidan extracted from *Sargassum glaucescens* acts as a natural antioxidant in the food industry, given that it exhibits dose-dependent antioxidant activities (Huang et al. 2016). Also, among the various forms which fucoidan takes in cancer therapy (Etman et al. 2020), the latest studies have proved their efficiency as coatings of magnetites in magnetic hyperthermia therapy with specific loss power values higher than either non-coated nanoparticles or other polymer-coated particles (Goncalves et al. 2021).

#### Laminarin

Laminarin is a linear polysaccharide that is mainly found in the glucan of Laminaria seaweed, made up of (1→6)- and (1→3)-β-glucose residues (Fig. 2) (Kraan 2012). Due to its antitumour, anticoagulant, anti-apoptotic, antioxidant, prebiotic, and anti-inflammatory qualities, laminarin is primarily used in the pharmaceutical and medical industries (Kadam et al. 2015; Pramanik et al. 2024). Laminarin primarily inhibits the growth of cancer cell colonies or the process of apoptosis to fight cancer and tumours (Zargarzadeh et al. 2020). To elaborate, human colon cancer (HT-29) cells’ ability to form colonies was prevented by laminarin from Alaria sp (Usoltseva et al. 2016). Also, in human hepatocellular carcinoma cell lines HepG2 and Bel-7404, laminarin from *Laminaria japonica* was demonstrated to be able to control the expression levels of senescence marker protein-30, hence preventing cancer cells (Tian et al. 2020). Laminarin exhibited potential prebiotic properties in addition to their antitumour effects since the digestive system’s hydrolytic enzymes do not hydrolyse them and are, therefore, very beneficial to the intestinal bacteria (Devillé et al. 2004; Deville et al. 2007). Adding laminarin as a supplement to a high-fat diet altered the gut microbiota, increasing Bacteroidetes while decreasing Firmicutes. As a result, the Firmicutes/Bacteroidetes ratio (F/B ratio) changed and shifted the microbiota towards higher energy metabolism, limiting the negative effects of the high-fat diet. This was done to develop anti-obesity functional foods (Nguyen et al. 2016). Laminarin was useful against viral plant diseases in agriculture (Meszka and Bielenin 2011) and aflatoxins (Hu et al. 2012). Laminarin may also act as a plant growth stimulant and raise plants’ tolerance to abiotic stress (Wu et al. 2016). Laminarin has other applications as well, including bioethanol generation (Motone et al. 2016; Mitsuya et al. 2017).

#### Ulvan

Ulvan is a part of the xylose, sulfated rhamnose, and uronic acid-based cell wall of *Ulva* sp (Lahaye and Robic 2007), supplying up to 45% of the dry weight of the algae with different chemical structures causing different morphologies of thallus (tubular and foliose) (Kidgell et al. 2024). Ulvan has a variety of applications in industries, including agriculture, functional foods, biomedicine, and pharmaceuticals (Alves et al. 2013; Venkatesan et al. 2015). The priming process is enhanced by ulvans from several *Ulva* species, which aids in plant defence (Kidgell et al. 2019). For instance, the *Arabidopsis thaliana* plant developed resistance to *Alternaria brassicicola and Colletotrichum higginsianum* fungi after being treated with ulvan from *Ulva fusca*(de Freitas et al. 2015). Ulvans may also play a part in the pharmaceutical and biomedical industries due to their anticoagulant, antioxidant, antitumour, and antihyperlipidemic activities, similar to those of other algal polysaccharides (Kidgell et al. 2019). The antioxidant activity of ulvan isolated from *Ulva pertusa* has been shown to demonstrate this point by increasing the enzymes catalase, glutathione peroxidase, and superoxide dismutase while decreasing malondialdehyde in a hyperlipidemic Kunming mice model (Li et al. 2018). Additionally, 100 g/ml of ulvan reduced the in-vitro viability of breast cancer, cervical cancer, and hepatocellular carcinoma cells to 0% (Ahmed and Ahmed 2014; Thanh et al. 2016). Ulvan from *Ulva linza* was also shown to have significant anticoagulant activity, which led to an increase of 3.3 to 6.2 folds in partial thromboplastin times compared to standard clotting times (Wang et al. 2013).

### Pigments

Algae, whether macro or micro, make vast sources of bioactive pigments that can be exploited for various applications (Patel et al. 2022). Algal pigments are summed as carotenoids, chlorophylls and phycobilins. Besides their primary role as antennas to capture light spectrum for photosynthesis, these pigments comprise antioxidative, anti-inflammatory, antitumour and other properties that make them valuable for biotechnological applications.

#### Carotenoids

**Fig. 3:**
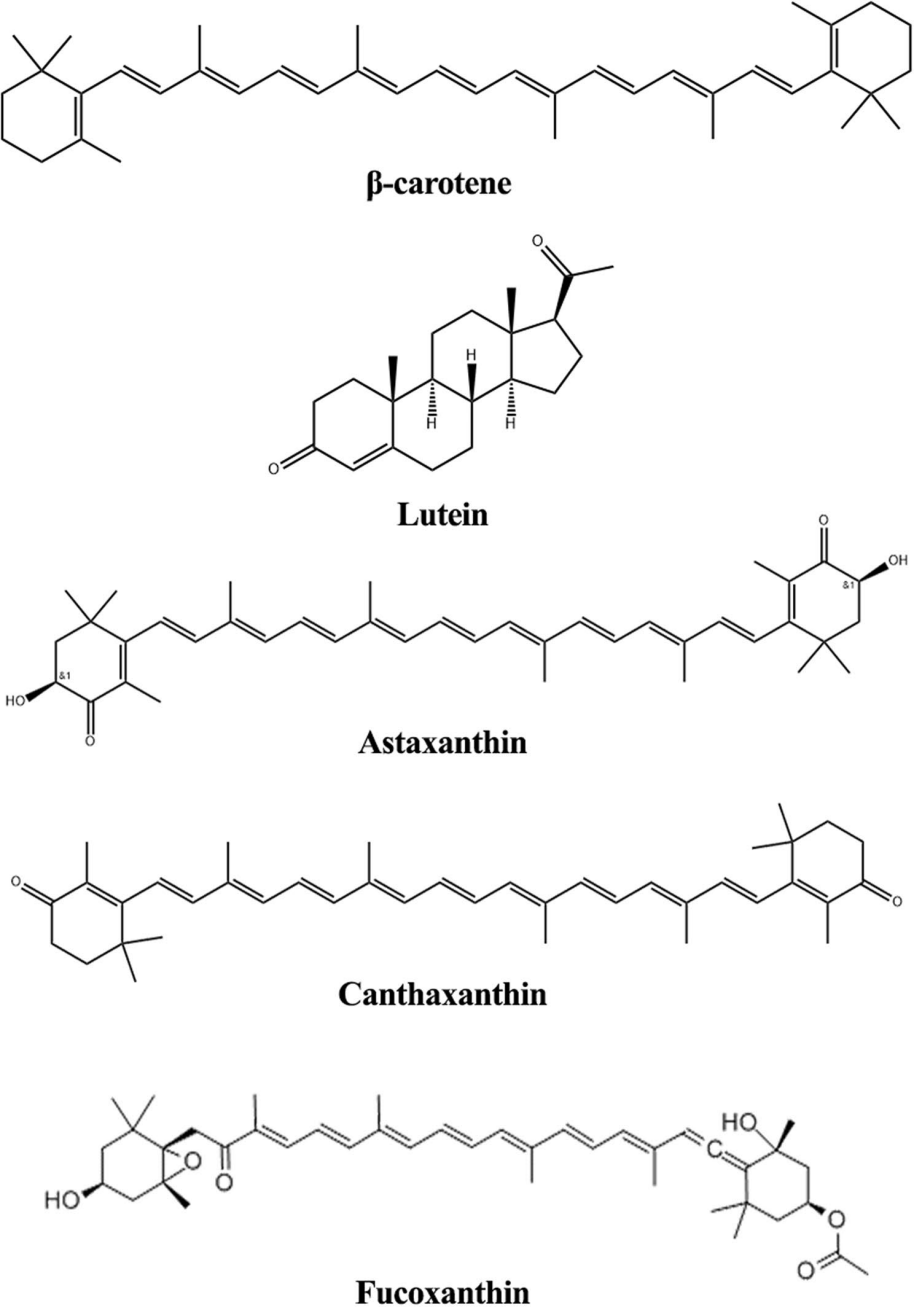
Chemical structures of carotenoids

Carotenes (α-carotene and β-carotene) and xanthophylls (lutein, astaxanthin, canthaxanthin, and fucoxanthin) are two subgroups of the group of lipid-soluble accessory pigments known as carotenoids (Fig. 3). They are present in diatoms, cyanophytes, dinoflagellates, cryptomonads, and chlorophytes (Cheng et al. 2020; Patel et al. 2022) can be induced by different stress conditions or plant hormones (Ma et al. 2018; Alsenani et al. 2019). Carotenoids derived from algae are utilised as additives, colourants, and supplements in food, feed, pharmaceuticals, antioxidants, anti-obesity agents, and cosmetic industries (Ambati et al. 2019; Kurniawan et al. 2023). Fucoxanthin from *Sargassum wightii* shows antioxidant and antihypertensive properties by inhibiting ACE (Raji et al. 2020). Additionally, carotenoids from *Neochloris oleoabundans* have shown antiproliferative action against human colon cancer cells (Castro-Puyana et al. 2017). Further, novel studies have considered the photosensitising ability of carotenoids and employed xanthophylls from *Cladophora* spp. as photosensitisers in dye-sensitiser solar cells (DSCs) (Lim et al. 2015).

#### Astaxanthin

Orange- red carotenoid pigment astaxanthin is mostly obtained from microalgae like *Haematococcus pluvialis* and *Chlorella zofingiensis*. It has garnered significant interest due to its strong antioxidant capabilities and wide variety of biotechnological uses. It has also gained significance in health supplements and medications due to its exceptional capacity to counteract free radicals and lower oxidative stress, which outperforms other carotenoids in their properties. For instance, astaxanthin lowers the risk of chronic illnesses including diabetes and cancer and improves cardiovascular health (Ambati et al. 2014). Also, its immunomodulatory effects have the potential to be used in the formation of nutraceuticals by boosting the immune response as they enhance antibody production and natural killer cell activity (Park et al. 2010). Furthermore, in models of Alzheimer’s disease, astaxanthin’s neuroprotective effects proved to be promising in controlling neurodegenerative illnesses by increasing cognitive performance and decreasing neuroinflammation (Fakhri et al. 2019). The antioxidant properties of astaxanthin are used in cosmetics to fight ageing and promote skin health. Topical treatments lessen wrinkles, increase skin elasticity, and shield the skin from UV ray damage (Seki et al. 2001). Astaxanthin’s potential as a natural sun protectant is highlighted by its capacity to protect against damaging UV radiation, minimising erythema and skin damage (Tominaga et al. 2017).

Astaxanthin is also used in animal feed to improve the colour of farmed fish and crustaceans, an important quality characteristic. In farmed fish, it enhances the immune system, reproductive efficiency, and survival rates (Elbahnaswy and Elshopakey 2024). Moreover, it improves antioxidant status, growth performance, and meat quality in poultry by darkening the skin and yolks of broilers and egg yolks (Gao et al. 2020). Biotechnological production of astaxanthin has seen significant progress lately. To maximise production, researchers are optimising cultivation conditions, for instance, by subjecting the algae to stress-induced conditions, such as high light intensity. (Nogueira et al. 2020). Meanwhile in order to develop a more effective and environmentally friendly manufacturing process, scientists are modifying important genes involved in biosynthesis using metabolic engineering tools like CRISPR-Cas9. (Kneip et al. 2024).

#### Chlorophylls

**Fig. 4:**
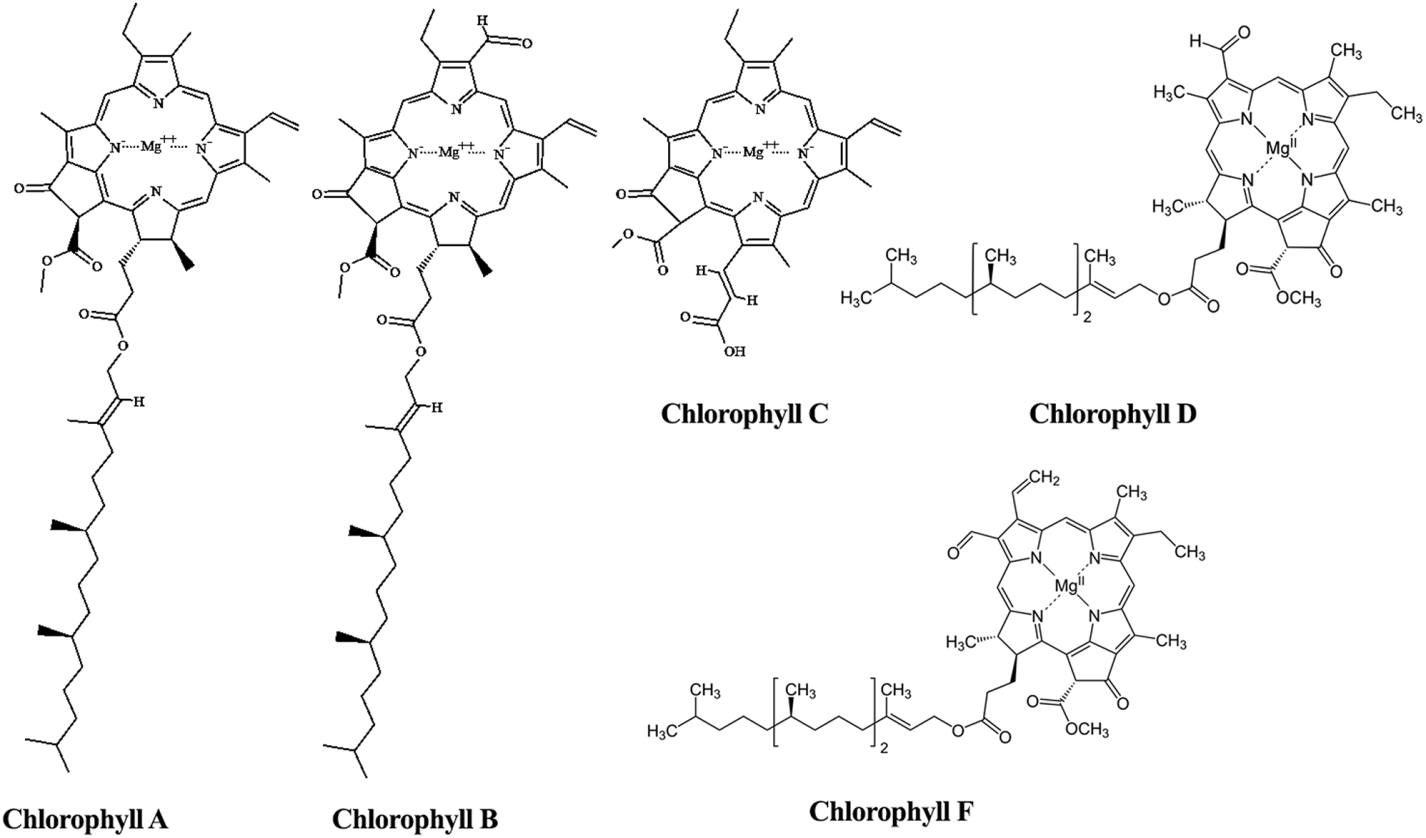
Chemical structures of chlorophylls

Chlorophyll is the main green pigment used in photosynthetic reactions by plants, algae, and cyanobacteria. They are branched into five types identified as chlorophyll a, b, c, d and f (Fig. 4) with absorption spectra 665, 652, 630, 696 and 707 nm, respectively (da Silva Ferreira and Sant’Anna 2017). Because of their stability, enhanced pigmentation, and antioxidant capabilities, chlorophylls are most frequently employed as natural colourants and additives in the food, nutraceutical, and cosmetic industries (Mourelle et al. 2017). With 1.5% of their biomass made up of chlorophyll, microalgae are among the top producers of this pigment (Dasgupta 2015). Various macro- and microalgae, such as *Ulva lactuca*, *Dunaliella salina*,* Chlorella vulgaris*,* Tetraselmis suecica*,* Botryococcus braunii*,* Ascophyllum nodosum*,* Fucus vesiculosus*,* and Postelsiapal maeformis*, have chlorophyll a, b, and c that has been used as an antioxidant and anti-inflammatory agent (Joshi et al. 2018). In addition, toothpaste and deodorants use chlorophyll from *Chlorella* sp. to mask any odours (Hosikian et al. 2010). A pancreatic cancer model showed an antiproliferative effect of chlorophyllin, a water-soluble component of *Spirulina platensis*(Konickova et al. 2014). Moreover, algal chlorophylls and carotenoids were formulated as shield materials against neutrons (Durna et al. 2023).

#### Phycobilins

Phycobilins/Phycobiliproteins (PBPs), a class of coloured fluorescent biliproteins found in certain microalgae, cyanobacteria, and Rhodophyta, which exist as phycocyanin, phycoerythrin, phycoerythrocyanin, and allophycocyanin. They can also be biosynthesised using bacteria, like *E. coli*, as expression systems. Phycobilins act as antennas that capture light energy in the 450–650 nm range and transfer it to the auxiliary photosynthetic complexes “phycobilisomes”. Normally the purification of phycobiliproteins require several steps, however, a new study developed a single step affinity purification method (Shi et al. 2024). Furthermore, they exhibit antitumour, antioxidant, antimetabolic and antimicrobial properties that make them promising for biopharmaceutical, biomedicine, and bioenergy applications (Chen et al. 2022; Dagnino-Leone et al. 2022). Furthermore, phycocyanin from *S. platensis* exhibited an antioxidative activity acting as a protective agent against liver damage caused by the oxidative stress induced by radiation by activating the *Nrf*2/ HO^− 1^ signalling pathway (Liu et al. 2020b). Also, phycobiliproteins peptides from *S. platensis* showed antidiabetic activity by inhibiting dipeptidyl-peptidase 4, an enzyme that destroys incretin and helps maintain the insulin-glucose balance in the body, with the value of half maximal inhibitory concentration (IC_50_) between 0.5 and 1.0 mg/mL (Li et al. 2020). Moreover, phycoerythrin and phycocyanin have shown considerable binding affinities to the SARS-CoV-2 proteases, thus inhibiting the virus (Pendyala et al. 2021). However, for their pigmentation and antioxidant properties, phycobilins are more commonly used in different industries such as food, pharmaceuticals, cosmetic colourants, and fluorescent detection systems (Vinothkanna and Sekar 2020).

### Minerals

Minerals are critical to maintaining national defence and economic security to promote the required high-tech industries. In 2018, the United States discovered 35 minerals that were added to the list of global minerals. Countries that possess many resources must improve the production of vital minerals that balance the depletion of environmental resources and governance capacity expansion. Between 1998 and 2021, many of these resources were depleted. The production rates for tin decreased by 61.43%, copper by 64.69%, and nickel by 74.77%. The metallurgical industry is exposed to many environmental pressures, so improving green total factor productivity (GTFP) production is essential for mining (Chen et al. 2020). It is well known that seaweeds have a remarkable capacity to synthesise the essential minerals and elements required for human diets, despite their levels varying greatly depending on their morphological properties, atmospheric conditions, and where they are located. Chlorophyta accumulates the highest concentrations of Mg, specifically Fe, whereas Phaeophyta and Rhodophyta accumulate the highest percentages of Mn and Me, respectively. Green seaweed tends to develop fewer Zn, Na, and K levels than brown and red seaweed. Due to the high mineral content of these algae, they can be used for various purposes, including as novel components for the production and enhancement of numerous functional dietary supplements. Many experiments have shown that seaweed can be used as an alternative to sodium chloride solution in ordinary food while enhancing its essential element content, which is sometimes lacking in European populations. When determining the proportion of seaweed utilisation, it has been demonstrated that excessive consumption of this element adversely impacts health. Studies revealed that despite its high biological accessibility, its level of bioavailability seems to be low, in contrast to elements such as Na, Fe, and K, where most algae have higher Na and K concentrations (Circuncisao et al. 2018). Sodium/potassium levels are often low (MacArtain et al. 2007). Additionally, seaweed contains hazardous metals such as arsenic (As), lead (Pb), cadmium (Cd), mercury (Hg) and copper (Cu) in differing concentrations that may be 200–500 times higher than those found in terrestrial plants. Therefore, marine herb intake can affect people’s health (Cardoso et al. 2014).

### Vitamins

Vitamins include numerous organic substances that serve as essential micronutrients. They involve a wide variety of biological functions, including hormones, coenzymes, regulators of cell signalling, antioxidants, and their control of cell and tissue growth or development (Inam et al. 2024). There are two major categories of vitamins: water-soluble and fat-soluble compounds. A, D, E, and K are the four types of fat-soluble vitamins. In contrast, vitamin C and the B vitamins [B1 (thiamine), B2 (riboflavin), B3 (niacin, which is nicotinic acid), B5 (pantothenic acid), B6 (pyridoxine), B7 (biotin), and B9 (folic acid)] are readily soluble in water. Most vitamins are produced by organisms that use sunlight to survive (Del Mondo et al. 2020). Vitamins’ functions in microalgae are essential as antioxidants, substrates or enzymes in the central metabolic reactions, control tissue growth and cell function. Specific vitamins, such as vitamin B7, have a few biological functions, while others play numerous functions, such as vitamin C. Microalgae manufacture vitamin A from provitamin A carotenoids (beta-carotene, beta-cryptoxanthin and alpha-carotene) (Toti et al. 2018). Vitamin E is also utilised as a source of antioxidants to combat photooxidative stress (Krieger-Liszkay and Trebst 2006). According to studies, tocopherol production in microalgae has been related to the synthesis of polyphenols in its reaction to abiotic stresses such as light, nutrients, or metallic substances (Goiris et al. 2015; Strejckova et al. 2019). Vitamin K1 is vital to plants, algae, mainly green algae, and certain cyanobacteria. It is an essential cofactor for redox reactions (Lefebvre-Legendre et al. 2007; van Oostende et al. 2008). At the same time, vitamin C, also known as ascorbic acid, is a precursor for numerous enzymes, plays a vital role in the biosynthesis of hormones and photosynthesis, and is essential for the recovery of antioxidants (Gallie 2013; Lisko et al. 2014). Moreover, ascorbic acid is vital in algae’s photoprotection and primarily in the photoprotective xanthophyll cycle (violaxanthin, diatoxanthin, antheraxanthin, and zeaxanthin) for the synthesis of lighting (Smerilli et al. 2017). Ascorbic acid regulates the quantity of hydrogen peroxide produced within the cell during photosynthesis by removing the hydrogen peroxide produced by the photo-absorption of oxygen in PSI (Photosystem I of Mehler reaction). The *vtc*2 gene (GDP-L-galactose phosphorylases) expression is enhanced. In contrast to plants, the initial step in L-ascorbate biosynthesis in *Chlamydomonas reinhardtii* occurs very quickly by hydrogen peroxide and a single molecule of oxygen, resulting in numerous enhancements in ascorbate content (Vidal-Meireles et al. 2017). Vitamin D primarily maintains critical calcium and phosphate levels in vertebrates’ skeletal structures by absorbing nutrients from the digestive system. Rickets, a softening disease of the bones in children caused by lack of vitamin D, has been linked to several other health conditions besides bone health, including immune disorders, heart disease, and some types of cancer (Hughes et al. 2018). Pigments and antioxidants have been extracted from microalgae recently by supercritical fluid extraction (SFE), which is one of the most necessary replacements for conventional organic solvent-based extraction techniques (Chronopoulou et al. 2019). Factors affecting extraction, including different temperatures, pressure, and adding of a polar co-solvent were studied on the quantities of carotenoids and fat-soluble vitamins extracted by SFE from *Tetradesmus obliquus* biomass.

### Polyunsaturated fatty acids

Polyunsaturated fatty acids (PUFAs) produced by microbes are gaining significant attention because they have many applications due to their vital health effects on organisms. Increased emphasis on product value for existing oily microbes or new microbes are being discovered through the implementation of new biotechnology strategies. Multidisciplinary approaches were considered, including high-throughput screening, metabolic engineering, and genome mining, and an emphasis was placed on co-cultivation and elicitation for the production of PUFAs (Liu et al. 2020a; Shah et al. 2022). They play a vital role in human health, including developing the nervous system, protecting the heart organs, and inflammatory disorders. Moreover, they also help reduce triglyceride concentrations in the blood and prevent cancer (Zarate et al. 2017).

These acids are found in plants, many animals and microorganisms, especially algae, fungi, and bacteria, and provide the highest diversity and superior alternatives for producing nutritionally essential polyunsaturated fatty acids (PUFAs) (Jovanovic et al. 2021). They can be classified into n-3 and n-6, which are essential to the health of humans and numerous other animals. They break down into lipid mediators such as resolvin and eicosanoids. It is vital to prevent the ineffective production of dietary plant PUFAs, as 20- or 22-carbon very long-chain PUFAs are used to form lipid intermediates and are, therefore, of increasing interest. Very long-chain PUFAs, which are taken in by animals and humans, eventually come from algae. Consequently, the production of algal lipids is essential for global ecological systems, as individual dietary fats, and as therapeutic agents against inflammatory diseases and obesity (Harwood 2019; Fang et al. 2023). In algae, the membrane components (glycosyl glycerides, betaine ether lipids, and phosphoglycerides) and storage lipids (tri glycerols) are the main lipids. In contrast, the percentages of polar membrane lipids vary greatly (Li-Beisson et al. 2019). However, these amounts typically contain a high concentration of PUFA. The total quantity of phytoplankton fatty acids was evaluated, and their location in various acyl lipids was also identified (Guschina and Harwood 2006; Kader and Delseny 2011; Li-Beisson et al. 2019).

### Peptides

Bioactive peptides are typically a class of biological molecules found in the molecular structure of parent proteins and perform their function following cleavage. An additional group of peptides is continuously produced and present in microscopic organisms. Various groups of bioactive peptides are marketed chemically or reconstituted. Bioactive peptides have many applications, including antimicrobial, antihypertensive, and antioxidant activities, lipid-lowering, anti-obesity, antidiabetic, and antiaging effects, and potency on mineral binding (Leong and Chang 2024). Algae are used as protein sources for balanced diets, and more recently, bioactive peptides have been discovered as algae-derived sources with potential health benefits. Bioactive peptides or cryptids are extracted from algae using (i) physical and biochemical methods for protein extraction and (ii) enzymatic hydrolysis and other techniques for producing bioactive substances. The biophysical characteristics of peptides extracted from phytoplankton employed for antihypertensive, antioxidant, and antiproliferative/cytotoxic activity have been described up to date. Several methods have been presented in silico for identifying peptides, including quantitative structure-activity relationships (QSAR) and molecular docking techniques (O’Connor et al. 2022). Bioactive peptides, also known as cryptids, are derived from algal proteins. These metabolites exhibit physiological effects once liberated from their inactive mother proteins (Sánchez and Vázquez 2017), like natural peptide hormones (e.g., those obtained from proopiomelanocortin, insulin, and angiotensin) (Harnedy and FitzGerald 2011). Many soluble peptide residues are exchanged with essential amino acid residues, such as lysine, which has enhanced antibacterial activity (Sato and Feix 2008; Almaaytah et al. 2014). The addition of alanine and lysine to the determined peptide increased its antibacterial activity without causing any damage to cells of eukaryotic species. PGWNQWFL and VEVLPPAEL are two peptides from *Navicula incerta* were used it to prevent the cytotoxic effect on HepG2/CYP2E1 cells, due to their antioxidant properties (Kang et al. 2012). A complex of nucleotides and peptides produced by *C. vulgaris* has the ability to repair damaged tissue, stimulate cell proliferation, and aid in the body’s recovery (Pradhan et al. 2014). Two peptide sequences Val-Ala-Phe, Ile-Ala-Pro-Gly, and Ile-Ala-Glu from *C. vulgaris* and *S. platensis* showed antihypertensive properties by maximally inhibiting the concentration of ACE-1 (Suetsuna and Chen 2001; Ko et al. 2012).

### Proteins

As a result of the population increase in other years and the existence of a society that is more aware of human health, the consumption of fats and carbohydrates was excessive, so the dietary protein intake increased with this population increase. To counter this, the solution to this problem is to increase the production of protein products by agriculture, aquaculture, and the food industry. Dietary proteins that are derived from animal sources are of the highest quality.

The solubility of microalgae proteins is pH-dependent; at pH values between 2 and 11, they were insoluble. Compared to whey and flaxseed proteins, the *Spirulina* sp. protein extraction’s oil-holding capacity (OHC) was superior. This extract was essential in nutritious foods such as meat. Algal protein isolates prevented angiotensin-converting enzyme I (ACE-I), and renin production can be used as heart-protective functional dietary ingredients and as alternatives to meat (Bleakley and Hayes 2021). Protein content was accurately measured in microalgae, shown to range from 7 to 40%, and this percentage can change dramatically over the life cycle of the algae. *Spirulina* algae contain a high percentage of protein (Saad et al. 2024b) which can supplement the human diet and is also used in preparing commercial products, including cosmetics and in many other applications (Pignolet et al. 2013; Templeton and Laurens 2015). A recent study showed that *S. platensis* supplemented with beet filter cake extract as a medium proved to be an efficient alternative for the production of single cell protein (Saad et al. 2023). Seaweeds are a promising choice because they produce much protein and have a low carbon footprint. They are also rich in the essential amino acids (EAAs) required for human consumption, but their nutritional value has not yet been compared to that of alternative protein sources using standardised measures. A new hybrid protein quality (HPQ) scale was used to calculate these scores after analysing the three species’ EAA content and protein digestibility. Seaweed proteins’ purity is similar to plant proteins such as soy, legumes, and other crops. Furthermore, seaweed proteins of various species have EAA profiles that are complementary and can be mixed to create protein variations that are nutritionally equivalent to milk and whey (Reynolds et al. 2022).

Moreover, seaweeds are considered an alternative and potential source of protein production. Using the rapid protein digestibility assay kit (k-PDCAAS) method, the amino acid values of *Palmaria palmata* had a score of 0.69 ± 0.014, although *Fucus serratus* and *Alaria esculenta* had scores of 0.63 ± 0.084 and 0.59 ± 0.021, respectively. Seaweeds have been researched to be rich in essential amino acids. These seaweed amino acid values indicate that this alga is an important alternative protein source that provides both essential and non-essential amino acids to the consumer for use in dietary supplements for humans (De Bhowmick and Hayes 2022).

### Polyphenols

Polyphenols (phenolic compounds) are among the most essential compounds from marine algae, which have a wide range of beneficial biological properties, including antioxidants, antimicrobials, antidiabetics, anti-inflammatory and anticancer agents, and include many different vital activities. These are secondary metabolites most prevalent in plants and included in some food sources. Although not considered essential nutrients, many of these compounds benefit human health. Polyphenols enter the phenol carbon ring, but they are formed in structures other than the carbon ring, and more than 500 different molecules have been discovered in food (Chiva-Blanch and Badimon 2017). They can be divided into two categories based on their chemical structure: compounds (flavonoids and non-flavonoids) and other subcategories. These compounds are increasingly used in food applications, cosmetics, and critical medicines. Other experimental studies have explored the antioxidant activities of polyphenols (Fernando et al. 2016). Researchers have examined the antimicrobial and antioxidant characteristics of naturally occurring marine *Cystoseira barbata* glycoconjugates (CBGs) to determine safe preservatives. CBGs have been isolated and chemically characterised to reveal that they contain sugars (49.76%), proteins (9.86%), and phenolic compounds (4.98%). Furthermore, when CBG was subjected to infrared spectroscopy to reveal the interactions between polyphenols, proteins, and sugars, the analysis confirmed that an alpha-type glycosidic bond and numerous sulfate groups in sugar residues were formed. The study of neutral sugars (CBGs) by gas chromatography-mass spectroscopy (GC-MS) showed that the conjugate sugars consist of galactose (34.02%), fucose (26.25%), mannitol (21.25%), a small amount of glucose (5.78%), and rhamnose (4.9%). Xylose (3.22%) and mannose (2.22%) are also present. The amino acid analysis of CBGs revealed an exceptionally high concentration of essential amino acids (40.36%). For the phenolic fraction of CBGs, qualitative tandem liquid chromatography quadrupole time of flight mass spectrometry (LC-QTOF-MS) was used as an alternative analysis method. This analysis revealed numerous phenolic compounds, such as flavonoids, glycosides, phlorotannins, and anthraquinones. CBGs showed various potent antioxidant activities, such as radical scavenging, chelating, and energy-reducing capabilities, as well as remarkable antibacterial and antifungal activities; this may pave the way for developing an algae-based natural conservation technique (Sellimi et al. 2017). Polyphenols from *Sargassum pallidum* showed potential anti Alzheimer’s and hypoglycemic activities (Jiang et al. 2024). Oxidation is a natural consequence of the metabolism of microorganisms, and this result is the formation of reactive nitrogen species (RNS) and harmful reactive oxygen species (ROS). Currently, many studies have discovered that polyphenols have very beneficial antioxidant properties. For example, *Hippophae* plants contain a high percentage of polyphenols and are used in many applications, including food, medicine, and cosmetics (Ji et al. 2020). In recent years, the isolation of bioactive components from seaweed has been fundamental worldwide. Experiments were conducted on various experiments on different types of algae, including brown algae, namely *Ecklonia cava*,* Eisenia arborea*,* Ecklonia stolonifera*, and *Eisenia bicycles*, because they contain potential biological activities that have the potential to act as an antioxidant, anti-inflammatory, antidiabetic, antitumour, and antihypertensive, as well as inhibiting the enzyme hyaluronidase and inhibiting the activity of matrix metalloproteinases (MMPs) (Thomas and Kim 2011). Also, they have many potential health benefits in functional foods, pharmaceuticals, and cosmetic applications (Wijesekara et al. 2010).

### Diterpenes

Diversely structured brown algae of the genus *Dictyota* are abundant sources of biologically active secondary metabolites. Significant discoveries have been made in the identification of diterpenes. Some diterpenes exhibit substantial biological activities, such as antiviral and cytotoxic drugs derived from diterpenes. The genus *Dictyota* is an abundant source of natural products with unimaginable pharmacological and biological characteristics. The detection of bioactive secondary metabolic products from species of the genus *Dictyota* has advanced significance (Bleakley and Hayes 2021). Most secondary metabolism products are diterpenes, with group II diterpenes (120 components) comprising nearly half of the diterpenes found in *Dictyota* species (233 compounds). A specific study on the genus *Dictyota* explained that the *D. dichotomy* is of the cosmopolitan type, in which all three diterpene groups (I, II, and III) produce diterpenes. From the *D. dichotomy*, 78 structurally unique diterpenes have been isolated. Due to their great pharmacological activity, many diterpenes are promising drug candidates (Reyes et al. 2004). The chemical examination of the organic molecules extracted from *Canistrocarpus cervicornis* has revealedtwo novel dolostone diterpenes 9 S-epoxy-14 S -4R-acetoxy-8 S, -hydroxy-7-oxodolastane (Bleakley and Hayes 2021) and 4R-hydroxy-8 S,9 S-epoxy-14 S-hydroxy-7-oxodolastane (Pignolet et al. 2013) and the dolostone that was previously isolated (4R,9 S,14 S)-4,9,14-trihydroxydolast-1(15),7-diene (Templeton and Laurens 2015) which is a necessary component of diterpene. An extensive spectroscopic analysis sheds light on the structures of the new chemicals. Compounds 1 and 3 were tested for cytotoxicity against cell lines of human prostate cancer cells (PC3) and human colon cancer (HT29). The results showed that the dolostone diterpenes (Reyes et al. 2004; Chen et al. 2018b) exhibit moderate concentration-dependent cytotoxicity (Campbell et al. 2017).

### Biofertilisers

Biofertilisation is a method of agriculture involving the use of a variety of biofertilisers that improve the soil’s nutrients while also enhancing crop productivity. Microorganisms were introduced to the soil to improve its properties, such as soil fertility and biomass productivity, which proved to be an active biological fertiliser that is both environment-friendly and pollution-free. Nitrogen-fixing cyanobacteria such as *Anabaena* sp., *Nostoc* sp., and *Oscillatoria anguustissima*, make an efficient cyanobacteria-based biofertilizer (Choudhary et al. 2024). Also, certain species of green microalgae and cyanobacteria (*Acutodesmus dimorphus*,* S. platensis*,* C. vulgaris*,* Scenedesmus dimorphus*,* Anabaena azolla*, and *Nostoc* sp.*)* have been utilised as successful biofertilisers to promote the growth of crops and alleviate drought stress (Ammar et al. 2022; Elnajar et al. 2024). For instance, *C. vulgaris* is considered the biggest and most common microalgae utilised in biofertiliser research. Furthermore, *Sargassum* sp. and *Gracilaria verrucosa*, lead to chemical changes as a signal to soil fertility in sandy and clay soils. Moreover, adding seaweed to the soil enhances it and increases its organic content, as well as returns the pH to the usual normal range and reduces the C / N ratio.

Waste and biomass extracts, generally, contain a substantial amount of nutrients that can be recycled using a variety of technologies and used to produce biofertilisers (González-González et al. 2019; de Siqueira Castro et al. 2020; Hussein et al. 2021). Comparing the production of phosphate biofertilisers derived from microalgal biomass to that of triple superphosphate, the Simpro^®^ system was utilised to analyse several environmental effects. Studies revealed that the functional unit for each fertiliser is 163 g phosphorous. Phosphorus was recovered from the liquid waste products of specific industries, including meat processing, the ponds of which contained high concentrations of algae. Biofertilisers produced from microalgae have lesssignificant environmental effects than conventional fertilisers. It was also established that all the energy used arises from the photovoltaic panels; consequently, the separation stage happens using a physical method that requires no energy consumption, so the biomass is dried in a dryer bed rather than the mechanical drying method. It turns out that the effect of biofertilisers is very similar to the effect of triple superphosphate. When the influences of substrate agriculture and the concentration stages are removed, the dehydration phase is the most significant because it contributes to the improvement of the biofertilisers’ benefit. Modern agriculture highly depends on synthetic chemicals, contributing to environmental pollution and soil depletion.

Microalgae and plant growth-promoting bacteria (PGPB) were considered alternatives to chemical fertilisers for improving soil fertility (Kang et al. 2021). This is due to its biofertiliser properties, which produce bioactive compounds (such as amino acids, plant hormones, and carotenoids), and its ability to inhibit pathogens that harm plants. Although a diagnosis that relies on one type of microalgae or bacteria is widely used in cultivation, experimental results indicate that a strong relationship between microalgae and bacteria may significantly affect the physiological and metabolic processes of each other synergistically. Therefore, the combined characteristics of microalgae and bacteria make them a promising biotechnological strategy for effective biomass production and sewage treatment. Many unexplored qualities remain regarding microbial interactions and microalgae for agricultural applications.

### Bioplastic

Waste resulting from plastic production is constantly rising worldwide, leading to pollution (Zeller et al. 2013; Onen Cinar et al. 2020). Thus, alternative biodegradable materials must be found to reduce this pollution. Recycling plastic pollutants alone cannot solve that critical problem. Therefore, bioplastics manufacturing from microalgae is a successful chance which should be investigated and enhanced. Additionally, bioplastic can be used in various applications, including cosmetics, food, and pharmaceutical packaging (Moreno-Garcia et al. 2017; You et al. 2017). In previous research, bioplastic material was generated from microalgae by two main approaches, mainly by mixing microalgae biomass, polymers, and biological additives or petroleum materials (Rahman and Miller 2017). Several techniques are used to produce these products, including thermo-mechanical methods. Other methods that depend on growth inside microalgae cells have been the production of biopolymers such as starch and polyhydroxybutyrate (PHBs). These products are then obtained and manufactured to create bioplastics; however, microalgae cells are not directly utilised. A mixture of polymer compounds and microalgae is designed (Onen Cinar et al. 2020). The microalgae-based bioplastics are processed by biorefining, and (Zeller et al. 2013) genetic engineering is used to produce biopolymer-producing microalgal strains. Much research explores the bioplastic production potential of microalgae similar to *Chlorella* and *Spirulina*, and they have microscopic cells, which makes both compounds desirable for bioplastic production (Zeller et al. 2013). Despite the significant resemblances between the two algae, *Spirulina* and *Chlorella* exhibit various actions and bioplastic properties when combined with polyethylene (PE) plastics due to their distinct amino acid compositions. The presence of compatibility devices improves the product properties of *Chlorella*-based bioplastics.

Also, the addition of 6% wt. of a compatibiliser to the mixture of *S. platensis* and polyvinyl alcohol (PVA) caused the formation of a bioplastic film with multiple properties, including higher tensile strength than commercial plastic bags. The conformer enhanced the plastic’s ability to stretch and allowed for smoother surfaces (Dianursanti et al. 2019). *S. platensis* was used as a reinforcement in plasticised wheat gluten in recent studies. Consequently, microalgae biomass particles with particle diameters greater than 5 μm lacked practical strengthening ability; smaller particles could interact with other compounds exceptionally effectively. *Scenedesmus obliquus* extract loaded on polyurethane constructs bioplastic films that have antibiotic properties (Abdo et al. 2024).

### Nanoparticles

Algae can act as nanoparticle biosynthesis factories, which is a distinctive feature due to the massive accumulation of metals inside the cell. Cyanobacteria such as *S. platensis* and *Aphanizomenon flos-aquae* synthesise Se, Pt, Au, and Ag nanoparticles. Since that was formed inside the cell, nanoparticles were released in media where they created stabled colloids to make it easy to use (Mukherjee et al. 2021). Nanoparticles have a large surface-to-volume ratio and can interact with other particles; thus, they gained high interest from researchers. Different methods, such as physical, chemical, and biological methods, produce silver nanoparticles. The biological method is considered the cleanest and safest one (Chugh et al. 2021; Saad et al. 2024a).

Using algae, organic and inorganic NPs (nanoparticles) can be prepared. Organic nanoparticles comprise chitosan (CS) and poly-ε-lysine, and quaternary polyelectrolytes and quaternary ammonium compounds. These organic molecules are less stable at high temperatures than inorganic molecules and inorganic NPs, such as antimicrobial polymers (Vincy et al. 2017). Chitosan nanoparticles have a wide spectrum of antimicrobial potency against many microorganisms, including bacteria, fungi, and viruses. They are non-toxic molecules that are biocompatible to human health and have the potential to act as absorption enhancers (Iqbal et al. 2020). Recently developed *Spirulina* based NPs possessed high mucoadhesive forces that makes it an efficient oral drug delivery system (Drori et al. 2024). Organic NPs production from algae has been a modern application in recent years due to the instability of organic NPs at high temperatures (Tiburu et al. 2017). The metal oxides of silver, zinc, iron (magnetite Fe_3_O_4_ and/or its oxidised form maghemite γ-Fe_2_O_3_), copper, and gold are the commonly studied inorganic NPs because of their unique electronic, catalytic, and optical characteristics (San and Shon 2018).

### Bioremediation and CO2 trapping

Bioremediation exploits the inherent capability of organisms to detoxify pollutants in an environment. Algae, due to their potential to absorb heavy metals and other pollutants from water and soil, play a vital role here. Many algal species, like *Chlorella* and *Spirulina*, have been reported to efficiently remove some heavy metals like cadmium, lead, and mercury from wastewater. These microalgae assimilate the pollutants into their biomass, which, on being harvested and processed, reduce environmental contamination (Laraib et al. 2020). Another avenue for sequestration is through algae as they are photosynthetic organisms that convert CO_2_ into oxygen and organic compounds. Therefore, this process can be harnessed for capturing CO_2_ from industrial sources using algal bioreactors. Some species, such as *Chlorella vulgaris*, demonstrate efficient CO_2_ capture and utilisation; under optimum conditions, as high as 70–90% (Razzak et al. 2013). Algal bioremediation coupled with CO_2_ trapping has numerous advantages including reducing greenhouse gas emissions, cleaning up polluted environments, and the produced biomass can further be used for a lot of applications like biofuel, animal feed, and biofertilizers, thus facilitating the transition to a circular economy. This would mean that algae perform the dual role of mitigating pollution and producing by products of value employing their potential for sustainable biotechnology (Mahlangu et al. 2024). However, some problems need to be tackled if this potential has to be realized which include reduction of production cost, increased efficiency of biomass collection, and improvement of the conditions necessary for growth. Genetic engineering and bioprocess optimisation are a means through which these challenges may be surmounted. The key aim of future research should be towards the development of robust algal strains that are able to cope with different environments, detoxify pollutants, and sequester CO_2_ fatty acids.

Algae are a potential source of energy-storing chemicals and important parts of their cell membranes. Their fats consist of saturated and unsaturated fatty acids of whichPUFAs are the most important for a variety of health benefits. Algae, particularly microalgae, are rich in several types of fatty acids, including omega-3 and omega-6 (Arora et al. 2021). The abundance and composition of fatty acids in algal species might differ significantly due to exposure to different environmental stressors and developmental circumstances.

### EPA and DHA

Long-chain omega-3 fatty acids, especially DHA and EPA, play a vital role in human health. Their main source comes from marine environments, of which algae is amongst the major. Microalgae, especially *Nannochloropsis* and *Schizochytrium*, are prominent sources of such bio-essential components since they naturally contain a very high amount of EPA and DHA (Spolaore et al. 2006). These lipids are very essential for brain development, cardiovascular health, and anti-inflammatory activity (Khavari et al. 2021). There are many well-known health advantages to EPA and DHA. For instance, they help to lower blood pressure, lower triglycerides, and lower the risk of heart disease. DHA is also an important structural element of the brain and retina, which is essential for seeing and cognitive function (Chen et al. 2023). Frequent consumption of these fatty acids has been associated with better immune disease management and decreased inflammation.

## Applications of algal toxins

Algal bioactive compounds, while medicinally useful, also consist of many toxins as well as protein inhibitors that are produced and secreted by the algae. These products, despite their potential toxicity, are extensively used in most clinical and biotechnological applications.

Microcystins produced by cyanobacteria have been identified to show remarkable antitumor activity (Zanchett and Oliveira-Filho 2013). To elaborate, microcystin-LR could cause the apoptosis of liver cancer cells; therefore, it can be a good potential agent against cancer. Likewise, nodularins are under research studies for their cytotoxic properties and application against cancer. Also, some algal toxins do have considerable antibacterial and antiviral properties (Llewellyn 2006). Cyanotoxins, including saxitoxin, have been studied in relation to their antiviral properties, and many different potential therapeutic applications are emerging regarding viral infections. Moreover, toxins from *Karenia brevis* act by binding to the voltage-gated sodium channels; such binding features make them very useful in the study of neurotransmission mechanisms in neural physiology and drug development for the treatment of neurological diseases (Llewellyn 2006). Recently, enormous research has been carried out on brevetoxins to elucidate neurodegeneration mechanisms and as neuroprotective drug leads. *Amphydinium* dinoflagellates produce ganyautoxins (GTXs), which are paralytic toxins that disrupt synaptic function by binding to voltage-gated sodium channels. Nonetheless, those biotoxins have proven to be a secure therapeutic method for treating either acute or persistent anal fissures (Garrido et al. 2005). GTXs act as painkillers by assisting in the relaxation of sphincters (Lattes et al. 2009). Due to its action as an inhibitor of protein phosphatase 2 A, okadaic acid from dinoflagellates, is employed in research to elaborate the methods by which conjugated linoleic acids may function as anti-tumor agents on breast cancer cells (Liu and Sidell 2005).

The algal toxins have also found uses in the development of biosensors for environmental analysis. For instance, saxitoxins and anatoxins have been used in the development of biosensors that are both sensitive and specific for the detection of the occurrence of harmful algal blooms in water bodies (McPartlin et al. 2017). The management of the water quality necessary for public health requires such biosensors.

## Limitations

Despite their diverse applications in nutraceuticals, cosmetics, pharmaceuticals, and food industries, algal bioactive compounds are faced with several limitations and challenges. This section elucidates these challenges, with a special focus on the co-extraction of toxic compounds, high production costs, strong flavours, and anti-nutritional factors, and proposes potential solutions.

### High production costs

One of the main hindrances in the exploitation of bioactive compounds from algae is the high cost of production. To elaborate, large-scale production is economically unfeasible due to the costs of cultivation, harvesting, and extraction processes. For instance, photobioreactors and advanced technologies used in the downstream processing of bioactive compounds escalate the overall expenses (Khan et al. 2018). Cost-efficient alternative cultivation systems like hybrid systems combining both natural light sources and photobioreactors, and open ponds are used to mitigate such costs (Slade and Bauen 2013). Furthermore, modifications by genetic engineering could augment the productivity of algal strains, reducing the total cost per unit of bioactive compound (Sharma et al. 2012). Emerging more efficient harvesting techniques, such as centrifugation, membrane filtration, and flocculation can as well lower expenses (Uduman et al. 2010).

### Co-extraction of toxic compounds

Some species of algae produce toxic substances such as phycotoxins and cyanotoxins that may be co-extracted along with the intended bioactive compounds. The difficulty is in finding selective extraction techniques that can effectively separate toxic from valuable substances. While complex purification techniques like chromatographic methods are essential, they also increase the extraction process’ complexity and expense (Herrero et al. 2010). Nevertheless, it is possible to enhance the purity of extracted substances through the application of selective extraction methods such as molecular imprinting polymers (MIPs) and supercritical fluid extraction (SFE) (Urriza-Arsuaga et al. 2023). Biotechnological approaches may also be applied in reducing toxin production in algae, metabolic engineering being one such innovative method (Wang et al. 2012).

### Strong flavors and odors

Another major disadvantage of algal bioactive compounds is related to their strong flavour and odour. These undesired qualities may reduce their appeal in food and cosmetic applications. For example, some algae-derived omega-3 fatty acids are foul-smelling, much like fish that users may find it unpleasant. Only in recent times has the need been presented for new strategies of formulation that could mask or eliminate unwanted flavour and odour (de Jesus Raposo et al. 2021). For instance, encapsulation techniques such as microencapsulation and nanoencapsulation can stabilise algal bioactive compounds and efficiently mask unpleasant flavours (Anal and Singh 2007). Further, for increased consumer acceptance, flavour-masking chemicals and even genetic manipulation of algae strains, that result in reduced amounts of the undesired flavours, can be utilised (Bleakley and Hayes 2017).

### Antinutritional factors

Antinutritional factors from algae, like caffeic acid, tannic acid, and gallic acid, may reduce the efficiency of nutrients by interfering with their digestion and absorption. In this respect, careful selection and processing with regard to algal species should be taken to ensure their efficacy and safety.(Singh et al. 2005). Processing techniques, from fermentation to enzymatic treatment, for the degradation of these compounds, might be effective in enhancing the nutritional quality of algal products (Jinek et al. 2012). Moreover, gene editing using CRISPR-Cas9 could also be applied to knock out undesired antinutritional factors.(Lam and Lee 2012).

## Conclusion

Algae are of great importance in producing biological materials of high economic value, which requires highly advanced methods in their production to reduce the cost of algal cultivation on a commercial scale and the development of various treatments to maximise the productivity of these added value compounds from algae. So, it is necessary, as much as possible, to think about how to apply modern scientific methods to grow algae on a large commercial scale in ways that combine maintaining the purity of the cultures, not contaminating them with undesirable contaminant organisms, and reducing the cost of production at the same time. Furthermore, more algae should also be certified by international food organisations as safe for human consumption and food, enhancing the biotechnological applications of algae on a broader scale. Also, many challenges are to be considered in the case of potential applications of algal toxins. Their production and purification can be complicated and expensive; at the same time, require very strict safety measures in their treatment and application. Future investigations should mainly be into the optimization of the methods of production, sensitization, and enhancing the specificity and efficiency of algal toxins in clinical and biotechnological applications in safe and sustainable environments.

## Data Availability

All the generated data figures are available as part of the article and no additional source data are required.

## Abbreviations

ACE: Angiotensin I-converting enzyme
AFA: Aphanizomenon flos-aquae,
Ala: Alanine
CBG: *Cystoseira barbata* glycoconjugates
CS: Comprise chitosan
CYP2E1: Cytochrome P450 Family 2 Subfamily E Member 1
DHA: Docosahexaenoic acid
DSCs: Dye-sensitiser solar cells
EAA: Essential amino acids
EPA: Eicosapentaenoic acid
GC-MS: Gas chromatography mass spectroscopy
GLA: Gamma-linolenic acid
Glu: Glutamic acid
Gly: Glycine
GTFP: Green total factor productivity
GTXs: Ganyautoxins
HepG2: Hepatoblastoma cell line
HPQ: Hybrid protein quality
IC_50_: Half maximal inhibitory concentration
Ile: Isoleucine
LC-QTOF-MS: Liquid chromatography quadrupole time of flight mass spectrometry
MIPs: Molecular imprinting polymers
MMPs: Matrix metalloproteinases
NPs: Nanoparticles
OHC: Oil-holding capacity
PDCAAS: Protein digestibility assay kit
PE: Polyethylene
PGPB: Plant growth-promoting bacteria
PHB: Polyhydroxybutyrate
Phe: Phenylalanine
Pro: Proline
PSI: Photosystem I
PSII: Photosystem II
PUFA: Polyunsaturated fatty acids
PVA: Polyvinyl alcohol
QSAR: Quantitative structure-activity relationships
RNS: Reactive nitrogen species
ROS: Reactive oxygen species
SFE: Supercritical fluid extraction
Val: Valine
β-PEA: Phenethylamine
